# Residual Lattice Strain and Phase Distribution in Ti-6Al-4V Produced by Electron Beam Melting

**DOI:** 10.3390/ma12040667

**Published:** 2019-02-23

**Authors:** Tuerdi Maimaitiyili, Robin Woracek, Magnus Neikter, Mirko Boin, Robert C. Wimpory, Robert Pederson, Markus Strobl, Michael Drakopoulos, Norbert Schäfer, Christina Bjerkén

**Affiliations:** 1 Photons for Engineering and Manufacturing Group, Paul Scherrer Institute, 5232 Villigen, Switzerland; 2 Department of Materials Science and Applied Mathematics, Malmö universitet, 20506 Malmö, Sweden; christina.bjerken@mau.se; 3 European Spallation Source ERIC, 22100 Lund, Sweden; Robin.Woracek@esss.se (R.W.); markus.strobl@psi.ch (M.S.); 4 Nuclear Physics Institute of the CAS, 250 68 Husinec—Řež, Czech Republic; 5 Division of Materials Science, Luleå University of Technology, 971 81 Luleå, Sweden; magnus.neikter@ltu.se; 6 Department of Microstructure and Residual Stress Analysis, Helmholtz-Zentrum Berlin für Materialien und Energie, 14109 Berlin, Germany; boin@helmholtz-berlin.de (M.B.); robert.wimpory@helmholtz-berlin.de (R.C.W.); 7 Department of Engineering Science, University West, 46132 Trollhättan, Sweden; robert.pederson@hv.se; 8 Neutron Imaging and Applied Materials Group, Paul Scherrer Institute, 5232 Villigen, Switzerland; 9 Imaging and Microscopy Group, Diamond Light Source Ltd., Oxfordshire OX11 0DE, UK; michael.drakopoulos@diamond.ac.uk; 10 Department of Nanoscale Structures and Microscopic Analysis, Helmholtz-Zentrum Berlin für Materialien und Energie, 14109 Berlin, Germany; norbert.schaefer@daimler.com

**Keywords:** residual stress/strain, electron beam melting, diffraction, Ti-6Al-4V, electron backscattered diffraction, X-ray diffraction

## Abstract

Residual stress/strain and microstructure used in additively manufactured material are strongly dependent on process parameter combination. With the aim to better understand and correlate process parameters used in electron beam melting (EBM) of Ti-6Al-4V with resulting phase distributions and residual stress/strains, extensive experimental work has been performed. A large number of polycrystalline Ti-6Al-4V specimens were produced with different optimized EBM process parameter combinations. These specimens were post-sequentially studied by using high-energy X-ray and neutron diffraction. In addition, visible light microscopy, scanning electron microscopy (SEM) and electron backscattered diffraction (EBSD) studies were performed and linked to the other findings. Results show that the influence of scan speed and offset focus on resulting residual strain in a fully dense sample was not significant. In contrast to some previous literature, a uniform α- and β-Ti phase distribution was found in all investigated specimens. Furthermore, no strong strain variations along the build direction with respect to the deposition were found. The magnitude of strain in α and β phase show some variations both in the build plane and along the build direction, which seemed to correlate with the size of the primary β grains. However, no relation was found between measured residual strains in α and β phase. Large primary β grains and texture appear to have a strong effect on X-ray based stress results with relatively small beam size, therefore it is suggested to use a large beam for representative bulk measurements and also to consider the prior β grain size in experimental planning, as well as for mathematical modelling.

## 1. Introduction

Titanium-based alloys have been widely used as engineering materials in many industries because of their excellent combination of a high strength/weight ratio and good corrosion resistance [1]. Application of the titanium and its alloys can be found in many different industries ranging from the aerospace to consumer goods, e.g., heat exchangers, automotive, offshore petroleum, gas exploration and medical implants [1]. Among various titanium alloys, Ti-6Al-4V is by far the most commonly used and accounting for some 50% of the total titanium output in the world [1]. However, extracting high purity titanium and the production of usable raw Ti-6Al-4V is a difficult and expensive process.

Many additive manufacturing (AM) techniques have been developed to fabricate geometrically complex and fully dense metal parts for a variety of applications. The most common AM methods use a laser or electron beams as the power source. In comparison with conventional methods, the electron beam melting (EBM) method has the advantage of increased component complexity with limited manufacturing expertise, shorter lead times, reduced material waste and minimum or almost zero tooling cost [2,3,4]. In the EBM, electromagnetic lenses are used for focusing and guiding of high-energy electron beams to selectively melting the ingredient powder to form a three-dimensional solid object point by point and layer-by-layer bases. The process is a fully computer controlled automatic system. Electron beam melted Ti-6Al-4V and its microstructure have previously been described by [3,4,5,6]. Despite the young age of the EBM process, it has already been demonstrated that it can create defect-free complex products with good mechanical properties, such as functionally graded cellular structures [7,8,9,10]. However, the EBM process is complex, and the results depend upon the variables of the system, such as beam power (beam current), beam size, scan speed, and scan direction/scan strategy. These are collectively referred to as process parameters. Thus, each set of process parameter settings produces a different built environment and cooling conditions, and as a consequence, different microstructures are observed [2,11] as well as possible different states of residual stress/strain (RS) and texture [12,13,14,15].

RS is internal, self-balanced stress that exists in alloy systems without any external applied forces and may appear during mechanical, thermal or thermochemical processing [16,17,18]. Depending on the compressive or tensile nature and magnitude of the RS, it significantly affects the mechanical properties of the materials. Lack of understanding RS distribution and the effect of different processing on the stress may create serious consequences [19]. Therefore, it is crucial to know the nature and magnitude of RS in the material for safe and economical operation. AM processes, such as EBM have already proven to be a potential manufacturing process in various fields with many possibilities. However, during AM processes, because of the repetitive selective point by point melting and solidification process, strong heat gradients are generated between different parts of the built material, which potentially can lead to unfavourable residual stresses. Despite several decades of development of AM processes, metal AM is still in its infancy and the relation between different AM process combinations with RS, microstructure, texture and material properties still lack significant understanding. Quality and performance of additively manufactured material can only be reliably controlled and optimized by understanding the effects of different process combinations on the RS and microstructure of the built material. There is limited research concerning RS on titanium alloys produced with conventional methods [17], wire/arc based additive manufacturing [20,21] and selective laser melting (SLM) [22,23,24]. However, there are only a few concerning RS in EBM Ti-6Al-4V [25,26,27]. Therefore, this study aims to explore the effect of a subset of EBM process parameters on residual strain/stress in Ti-6Al-4V. RS can be investigated by e.g., hole drilling techniques, ultrasonic and magnetic methods and diffraction-based methods [16,18,28]. Each RS measurement technique has its own advantages and disadvantages. Among them, diffraction (e.g., X-ray and neutron diffraction) is one of the most accurate and well-developed methods of quantifying local and global residual stresses in the material [16,18]. Compared with conventional RS measurement techniques, the diffraction method offers many advantages. The residual stresses in the material are calculated from the strain measured in the crystal lattice. Generally, RS diffraction measurements are not significantly influenced by material properties such as hardness, the degree of cold work, or preferred orientation. In fact, all can be quantified by diffraction techniques. In addition, they are non-destructive, have a high spatial resolution (ranging in the order if μm to mm depending on the radiation source)), almost no specimen geometry restrictions, and can be applied to all types of crystalline material. Furthermore, with the high-energy synchrotron X-ray diffraction setup used in this work, it is possible to characterize and conduct quantitative studies on multi-component systems containing phase quantities as low as 0.1–0.7% [29,30,31] with a high temporal resolution (0.2 s) [31]. This means that phase specific RS in the target system can be determined dynamically with high accuracy. Last but not the least, diffraction-based RS techniques are already well established—there is good level of expertise, and standards have been developed [16,18,32].

To investigate the effect of EBM process parameters on residual strain and stress development, various specimens produced with different beam sizes, scan speeds and build thicknesses were investigated. In addition, original powder specimens that were used to produce these solid specimens have also been tested. Diffraction measurements were carried out using the high energy (50–150 KeV) beamline I12-JEEP (joint engineering, environment and processing) at the Diamond Light Source (UK) with an energy dispersive X-ray diffraction (EDXRD) setup [33], as well as the dedicated RS neutron diffractometer (ND) E3 [34] at Helmholtz Zentrum Berlin (Germany). After data collection, the Pawley pattern fitting method was performed for the EDXRD data and single peak fitting for the ND data using the structure analysis software package TOPAS-Academic [35].

## 2. Experimental Setup and Data Analysis

### 2.1. EBM Process and Material

The EBM process is described in greater detail e.g., in [36]. However, the EBM process is quite complicated owing to numerous parameters (e.g., feed material, beam current, feed rate, build layer thickness and scan speed), which potentially change the build environment and cause a different micro-structure in the final product that may not be wanted. The solidification rate, surface smoothness, and microstructural homogeneity of EBM processed parts are strongly influenced by the process parameters.

The electron beam parameters, such as beam speed, beam current and scan length can be varied in a controlled sequence throughout the build according to algorithms developed by the manufacturer. According to the literature, the resolution of the build in EBM is influenced by layer thickness [37,38] powder size [39] and spot size [2]. It has been reported that the size of the powder and layer thickness has a direct influence on build part quality [38,39]. Commonly, a smaller powder gives finer surface finish and it has been demonstrated that the EBM can process powder with size 25–45 μm [39]. The benefit with smaller particles is finer surface finish and more compact layer i.e., no/little shrinkage. Nonetheless, due to the charging from the electron beam, smaller powder particles are not possible for EBM i.e., a phenomenon called smoking can otherwise occur. A benefit with larger particle size and layer thickness, however, is an increased build rate. To achieve the highest quality, the current standard EBM layer thickness has been reduced from 100 to 50–70 μm and powder with size 45–100 μm was used [40]. The powder used in this study was gas atomized and size within the range of 45–100 μm. The chemical composition is Ti-6Al-4V-0.1Fe-0.15O-0.01N-0.003H (in wt %).

The mechanical properties of the EBM built Ti-6Al-4V are affected by scan speed [41]. It has been reported that the rapid heating and cooling, and repetitive thermal cycles of AM processes in laser-based additively manufactured material create RS in as-built material [42,43]. According to Klingbeil et al. [43], preheating of the build platform and powder layer can reduce RS and negative effects, such as part warping. In the EBM process, for Ti-6Al-4V the whole build chamber is heated to approximately 700 °C prior to building, and each layer of powder is preheated before melting. The minimum temperature of the build chamber is maintained ≥ 600 °C throughout the build process [40]. Therefore, the EBM built component is naturally annealed immediately after building, and thus remaining residual stresses may be limited in magnitude. However, the relation between scan speed, offset focus and RS has not yet been verified experimentally to the authors’ knowledge.

**Figure 1:**
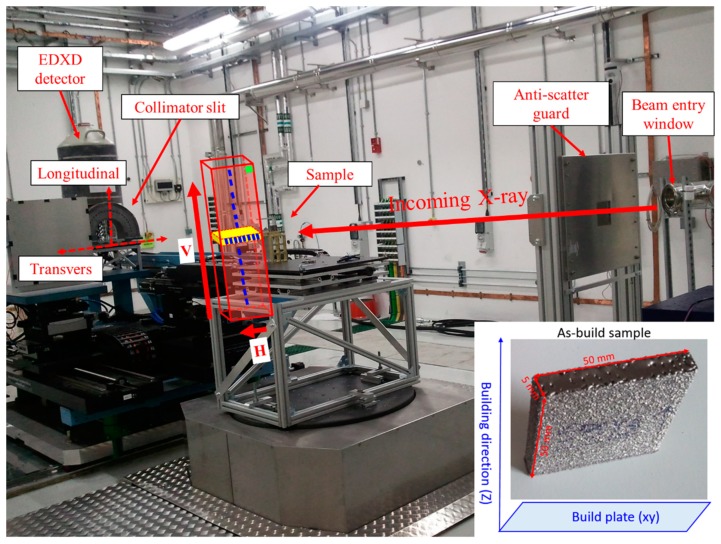
Beam line setup and illustration of measurements. “V” is the vertical direction and “H” is horizontal direction. Each blue and green rectangle represents the measurement locations. Embedded image is a photo of one of the as-built specimens.

Ti-6Al-4V blocks of 50 mm × 50 mm × 5 mm were built and measured at the centre of each specimen both along the build direction (vertical, “V”) and in the build plane (horizontal, “H”) by neutron diffraction. To investigate the finger prints of the phase specific residual stresses, specimens were cut from the as-build blocks in varied thicknesses (see Table 1) and investigated with synchrotron XRD in two directions (Figure 1). For the analysis of the RS state and microstructure, three different standard controlling factors were chosen: build thickness, scan speed and offset focus. The details of specimen production parameters are tabulated in Table 1. Three specimens were made for each set of processing parameters. Because of limited beam time, only one specimen was measured during neutron diffraction experiment.

### 2.2. Synchrotron X-ray Measurements (EDXRD)

Synchrotron X-ray diffraction is a well-known and powerful tool for structural analysis, quality characterization [18,44] and conducting quantitative studies on multi-component systems [30,31]. Herein reported experiments were performed at the high energy (50–150 KeV) beamline I12-JEEP at Diamond Light Source in the UK with EDXRD setup [33]. All specimens were irradiated with a white/continuous X-ray beam with a photon flux range from 1.8 × 10^11^ to 9.4 × 10^10^ photons s^−1^ depending on the energy of the X-ray beam. The EDXRD data were recorded with a “horseshoe” detector, consisting of 23 liquid-nitrogen-cooled germanium (Ge) energy-sensitive detector elements, positioned 2 m behind the specimen position with a take-off angle of 5° from the incident beam. The energy resolution of Ge-detector ranges from 7 × 10^−3^ at 50 keV to 4 × 10^−3^ at 150 keV. As the 23 detector elements are equally spaced in steps of 8.2°, they have full azimuthal coverage from 0° to 180°. During the measurements, each detector element independently records diffraction patterns under a different Bragg angle. As the sequence of energy values of each detector element is approximately the same for all, the 23 diffraction patterns can be summed to form one pattern after normalization of each data set for detailed phase analysis. The actual EDXRD setup with schematics of measurements and specimen can be seen in Figure 1.

In the experiment, a 2-mm thick copper filter and slit sizes of 0.1 × 0.1 mm^2^ and acquisition time of 100 s were used. To map strain variations in the material along build direction and in the build plane as complete as possible in the limited synchrotron beam time, measurements were made at an average of 55 points in the centre of the specimen along the vertical, and 10 in the horizontal direction. The volume of the material contributing to the diffraction pattern corresponds to the intersection of the incident and diffracted beams, typically defined by slits and collimators, respectively. To ensure the gauge volume is fully inside the specimen, the centre of the measurements points close to the edges was positioned about 300 μm from the edge during horizontal and vertical line scans in the centre of the specimen. The clearance of corner scan, however, is about 0.5 mm from the corner edge. Each detector element in the setup measures the lattice spacing in a specific direction, which means that strains in 23 different directions in 0–180° range can be measured in a single measurement without specimen rotation. The measured strain direction can be defined by the X-ray scattering vector, which bisects the incident and diffracted beams. With this EDXRD setup, the three-dimensional strain/stress field can be calculated by including additional information or by adopting assumptions, such as the plane strain criterion. For that reason, only strains along the longitudinal and transversal directions are presented in this work. Detector elements 1 and 23 were used to measure transverse strain, and elements #11 or #12 were used to measure longitudinal strain. Compared with the angle dispersive diffraction setup used in the lab X-ray source, the JEEP (I12) at Diamond has many orders of magnitude higher X-ray flux with high energy. At JEEP it is possible to probe metal specimens in millimeter to centimeter thickness in relatively short times. This gives the possibility to determine the RS state of the various phases in one experiment. This was important for the present study as thicker specimens allowed to obtain better statistical average (larger diffracting volume, i.e., more grains) for proper RS state probing.

### 2.3. Neutron Diffraction Studies

To avoid setup or instrument dependent error and justify the observations, the same specimens were investigated with the neutron diffractometer E3 [34] and the ESS test beam line V20 [45,46] at the research reactor BER-II in the Helmholtz-Zentrum Berlin (HZB). E3 is a constant wavelength and angular dispersive instrument (detector is moved around 2θ), whereas V20 is a time of flight beamline, where a wavelength dispersive setup with a fixed detector position was used.

### 2.4. Microstructure Studies

All specimens used for microscopic studies were prepared by using standard metallographic preparation routines. To investigate the microstructure of the specimens, visible light microscope (Nikon ECLIPSE L150, Amsterdam, The Netherlands) and scanning electron microscope (SEM, ZEISS EVO LS10, Zeiss, Oberkochen, Germany) were used. The SEM was operated using an acceleration voltage of 25 keV with magnifications ranging between 1000 and 5000×. Electron backscatter diffraction (EBSD) scans were performed in an SEM equipped with a field emission source and an automated EBSD acquisition system. Here, the SEM was operated at an accelerating voltage of 20 kV and a beam current of 5 nA. The EBSD scans were acquired with the specimen tilted to 70° at a working distance of 10 mm. Two EBSD maps have been obtained from two different locations at the centre of the specimen along the build direction.

### 2.5. Diffraction Data Analysis

After measurement, the software MATLAB was used to extract the data in xy binary format, which is friendly to many structure refinement programs. Then, the structure analysis software packages TOPAS-Academic was used to carry out least-square refinement (Pawley method) and standard single peak fitting routines [35] for stress/strain analysis. Phase quantities of the sample were determined by the Rietveld analysis [35]. The basic crystal structure information of the various phases needed for the Pawley and the Rietveld refinement were obtained from the Inorganic Crystal Structure Database (ICSD) [47]. All structural data used, such as reference and refined parameters, are tabulated in Table 2.

As overall diffraction patterns (or peak shapes) are a convolution of background, specimen and instrument, a standard cerium oxide specimen with lattice parameters 5.41165(1) Å was measured under the same configurations for calibration and instrument profile function extraction purpose. Then, the Pawley [35] refinements were performed to determine the profile function. After a good fit was obtained from cerium oxide data, the instrument profile function was set fixed together with zero error correction, and then a Pawley batch fitting was carried out to refine the structure of various phases to define best possible unit cell parameters of each measurement. The Pawley fitting was performed on the full diffractogram, which spans from 0 to 145 degrees in 2θ. The peak profiles were modelled with a modified Thompson–Cox–Hastings pseudo-Voigt (pV-TCHZ) profile function [35]. The background was fitted with a Chebyshev function with six coefficients and the zero-shift error together with axial divergence calibrated with a standard cerium oxide reference specimen. During batch refinement all instrument related parameters were kept fixed and only unit cell parameters together with background were refined. To avoid any complication introduced by oxides, elemental composition variation, and phase distribution to the measured strain, a single peak fitting was also performed together with the Pawley fitting in limited diffraction ranges, i.e., 1.19639–1.79480 Å.

### 2.6. Strain Calculations

Diffraction patterns are routinely used as a fingerprint of a material’s crystalline structure. Any external stimulus, such as applied load or heat gradient can alter the interplanar lattice (*d*) spacing of the material, causing a change of the diffraction pattern. Therefore, the atomic lattice spacing in crystalline materials can be used as a natural strain gauge [16,48]. For instance, tensile stress will cause an increase of the lattice spacing in a given direction and compressive stress leads to the opposite. The average elastic lattice macrostrain (*ε_hkl_*) in the sampled volume can be calculated by comparing the measured lattice spacing *d_hkl_* with that of the unstrained (stress-free) lattice spacing *d*^0^*_hkl_* as [16,48]:

(1) $$\varepsilon_{hkl}=\frac{d_{hkl}-d_{hkl}^{0}}{d_{hkl}^{0}}$$

If the target material is isotropic and all grains/domains in the material have the same responses to external stimulus, then the Equation (1) is sufficient to determine the stress/strain state in the material for a given direction of the stress/strain tensor. However, most crystalline materials have anisotropic properties, including the stiffness of the unit cell, and hence accurate stress/strain analysis requires consideration of multiple reflections. While individual peak fitting can be used to investigate the elastic and plastic anisotropy of individual lattice plane families, a fit of a complete diffraction profile—as used for results reported herein—can be used to determine the average lattice parameter from which the strain can be calculated that is closely representative of the bulk macroscopic strain (for the same direction).

The strain in a cubic material/phase (e.g., β-Ti) can be calculated by [48,49]:

(2) $$\varepsilon_{cubic}=\frac{a-a_{0}}{a_{0}}$$

Similarly, the strain in a hexagonal material can be calculated by [48,49]:

(3) $$\varepsilon_{hexagonal}=\frac{2\left(\frac{a}{a_{0}}-1\right)+\left(\frac{c}{c_{0}}-1\right)}{3}$$

where *a* and *c* are the lattice parameter of the given phase; *a*_0_ and *c*_0_ are the strain-free lattice parameters. Unstrained lattice parameter can be extracted by several methods, e.g., summarized by Withers et al. [32]. In this work, the $d_{hkl}^{0}$, *a*_0_ and *c*_0_ are obtained by (1) measuring the raw powder used for building these specimens used in the study; (2) measuring from the corner of each specimen; (3) taking the mean values of all scans of each sample.

## 3. Results and Discussion

### 3.1. Microstructure

**Figure 2:**
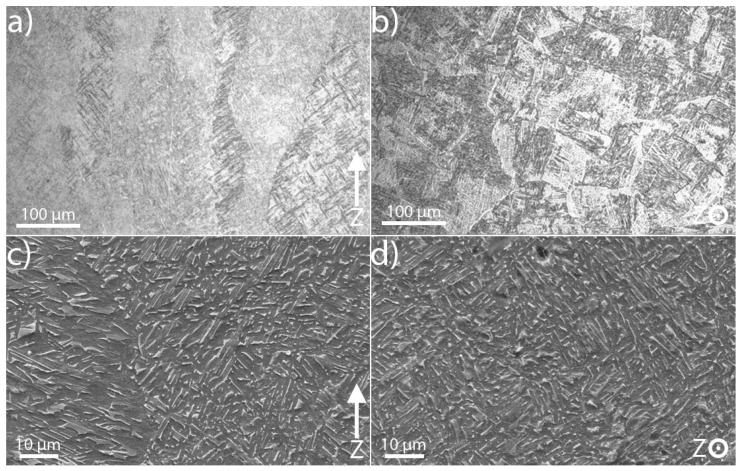
Light optical microscopy image of S7V (**a**), and S7H (**b**). In (**c**) and (**d**), the images are taken with a SEM where the specimens used are S8V and S8H respectively. Z indicates the build direction and etching procedure employed to obtain the alloy microstructures was by immersion in Kroll’s etchant for 10 s.

Depending on processing and alloying elements, the Ti-6Al-4V microstructure may include α-Ti (hcp), β-Ti (bcc), α′ (hcp martensite), as well as different amount of dislocations, substructures and twinning [1,4]. The EBM built Ti-6Al-4V, however, commonly consists of two phases, namely α-Ti (hcp) and β-Ti (bcc) twinning [2,4,5,50]. Typical visible light micrographs of one of the specimens from build-direction and build plane are shown in Figure 2a,b, respectively. All materials studied show a microstructure which consists of intertwined α lath colonies where individual α-laths are separated by a thin layer of retained β-phase. This type of microstructure is formed when fast cooling rates from the β-phase field are achieved [51], the presence of grain boundary α does, however, indicate that the cooling rate was not fast enough to suppress the grain boundary α nucleation, which can occur for AM processes with even faster cooling rates. High-resolution SEM micrographs corresponding to Figure 2a,b are shown in Figure 2c,d, respectively. In these SEM micrographs, the dark colour corresponds to the alpha phase and white colour corresponds to the β phase. The microstructure shown in Figure 2 is similar to the microstructure of EBM built Ti-6Al-4V reported by others [2,3,4,11,15]. The columnar nature of prior β grains that is shown in Figure 2 is a direct consequence of the thermal gradient that exists in the build direction [15].

**Figure 3:**
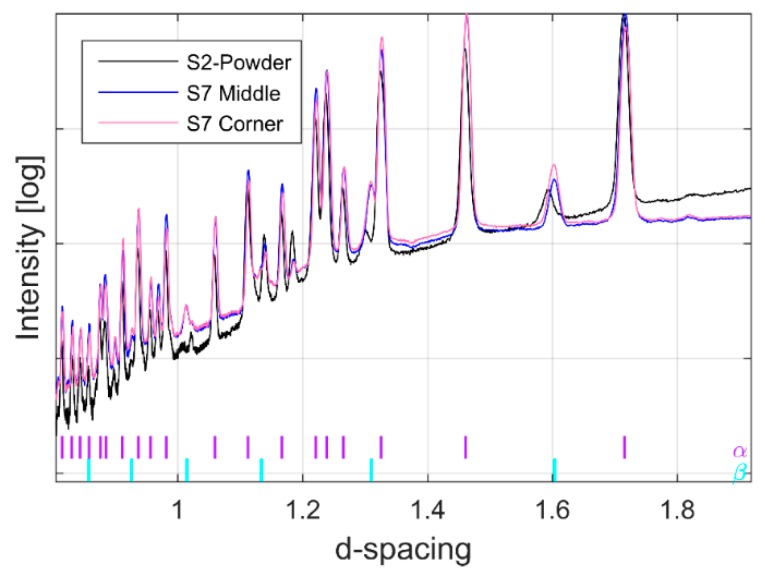
Diffraction pattern obtained by EDXRD of Ti-6Al-4V powder and EBM built material. The colour coded, small vertical tick marks represent the hkl peak positions of labelled phases in the same colour (α = purple, β = magenta).

Synchrotron X-ray and neutron diffraction work confirmed the α+β microstructure observed by microscopy. Figure 3 shows the raw XRD diffraction patterns of as-received Ti-6Al-4V powders used for building all the specimens investigated in this study together with a representative diffraction pattern of EBM built material collected at the centre and one of the top corners (close to the final layer) of the same specimen. The colour coded, small vertical tick marks in Figure 3 (and all other figures throughout) represent the hkl peak positions of labelled phases in the same colour. As expected, the diffraction patterns from the corner and centre of the built material showed close similarity, but they both differ significantly from the powder pattern. According to Figure 3, it is evident that the as-received Ti-6Al-4V powders and the EBM specimens (independent of build location) contain predominantly α-phase with the HCP structure, consistent with previous studies [52]. In addition, the system also contained detectable amount of BCC β-phase (1.5–10 wt %) together with limited quantity of Ti-oxide (<1 wt %). The peak position and the intensity of the oxide diffraction peaks measured at different parts of the specimen did not show observable variation. Therefore, the effect of the oxide is excluded from the discussion. The presence of the β-phase can be clearly seen by the corresponding high angle diffraction peaks at d-spacing = 2.26 Å and d-spacing = 1.60 Å for the (011) and (002) reflections as reported in [53,54].

It is known that the mechanical properties of the Ti-alloys strongly depend on the distribution of alloying elements and phases present in the system [1]. Generally, Ti-alloys show higher strength and higher density but lower creep strength with an increase in β-Ti phase content [1]. To ensure and control the properties of the material for a specific application, it is important to understand how different material processing affect the material phase composition of the alloy system. The microstructure and mechanical properties of Ti-6Al-4V for specific applications can be tailored by post heat treatment after manufacturing. Commonly, the post thermal process operations will take place in the α+β phase and/or single β-phase field. The volume fraction of β-phase increases with temperature and therefore governs the mechanical properties [55]. Therefore, it is important to understand how different AM processes will influence the formation/distribution of the β-phase in the built material. However, because of the limited phase quantity at room temperature of the β-phase in Ti-6Al-4V and its low diffraction strength, the β-phase has not been observed [56] or has been excluded from the analysis. While studying Ti-6Al-4V fabricated by the selective laser melting process (SLM), Chen et al. [56] did not observe a clear β-phase from either the as-received powder or the built solid specimens from their XRD work. According to Chen et al. [56], the reason behind weak or absent β-phase in their system is the high cooling rate used during the build. They argued that [56], SLM processed specimens maintain the original composition and crystal structure of the powders; all possible existing β-phase at high temperatures might all transform into the α or martensitic α’ phase via rapid solidification during the SLM process. However, it is interesting that even the as received Ti-6Al-4V powder used in their work did not show detectable β-phase. We think that such phase absence might be explained by the sensitivity of the conventional XRD method and the textures present in the system in addition to the rapid cooling of SLM processing and powder manufacturing.

**Figure 4:**
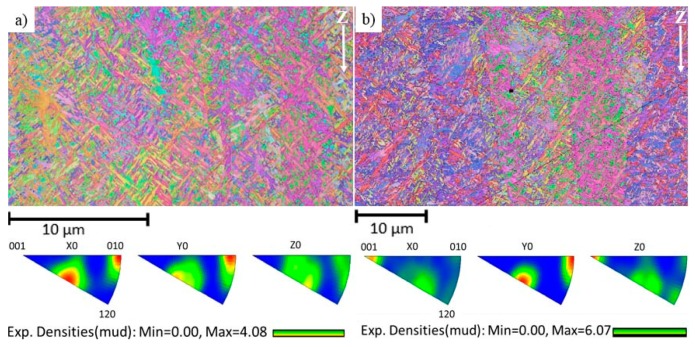
Inverse pole figure maps of Ti-6Al-4V (S7V). The build direction is indicated by the white arrows. (**a**) is close to the last layer; (**b**) few mm below from (**a**); Images under (a,b) is the corresponding inverse pole figures.

Ti-6Al-4V has been shown to have texture [1,12], and its development strongly depends on processing type and temperature [55]. Because of localized melting and solidification together with repetitive heating processes, the microstructure and texture formed in additively manufactured material are different from that in cast or wrought materials. In order to emphasize the complexity and variation in textures in EBM built Ti-6Al-4V, two grain orientation maps together with corresponding inverse pole figures obtained from the same specimen measured along the build direction at two different areas in the specimen are shown in Figure 4. Figure 4a is close to the last melted layer and Figure 4b is a few mm below. As can be seen in the figure, the material shows preferred grain orientation (texture), and it varies from one location to another despite that these two measurements were done in the same vertical plane along the same line and only a few mm apart. Microstructure and textures presented in Figure 2 and Figure 4 indicate the complexity of the Ti-6Al-4V microstructure produced by EBM. Because of this highly varying microstructure and its strong correlation with the AM process parameters and build geometry, the mechanical properties of the material reported in the literature shows a large scatter [5,50,52].

### 3.2. Peak Intensities and Phase Compositions

**Figure 5:**
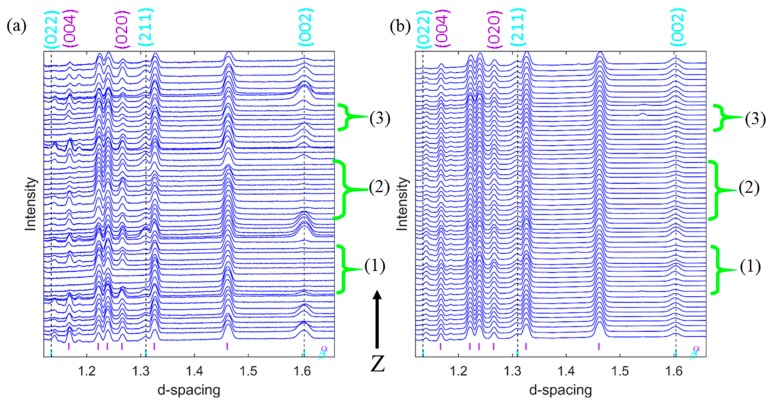
Assembled diffraction patterns of a vertical scan (along the build length) of one of the EBM Ti-6Al-4V samples (S7V). (**a**) is from the detector element 15 alone and (**b**) is the sum of all 23 elements of the same scan. The black arrow indicates the build direction from bottom to top.

All observed diffraction patterns from one diffraction angle (from detector element 15) collected during a vertical scan of one of the specimens, and the corresponding specimen patterns obtained from all 23 directions from the same specimen are presented in Figure 5a,b, respectively. As shown in Figure 5a, some of the α- and β-phase diffraction peaks are absent in certain parts of the build material from the diffraction patterns collected from only one scattering direction. From Figure 5a, one can see that there is no correlation between the absences of α- and β-phase diffraction peaks. In addition, such an absence does not coincide with the additive build layer thickness. Similar observations have also been observed for all other specimens studied in this work. The summed diffraction patterns of all measurements in all 23 scattering directions, however, showed all corresponding α- and β-phase diffraction peaks with similar peak intensity and width with a sign of α- and β-phases homogeneity as shown in Figure 5b. This difference is thought to be a result from the preferred orientation of the different grains (i.e., the α-phase is also present in probed volumes that do not show any α-peak for a single detector, but the peaks are observed in other scattering angles from the same volume). In Figure 5, HKL indices of α- and β-phases with significant changes are given at corresponding peak Bragg peak positions and interesting regions where β-phase peaks are absent in detector element 15 also marked with (1), (2) and (3) with green curly brackets.

**Figure 6:**
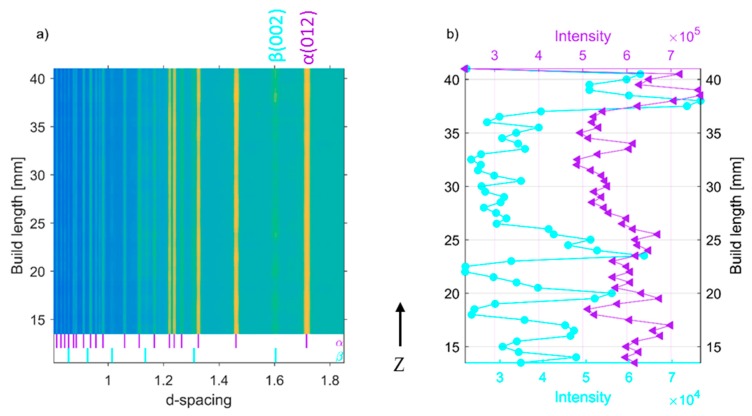
(**a**) Accumulated diffraction patterns of vertical line scan along the build direction of specimen S8, and (**b**) corresponding peak intensity of (002) β-Ti (magenta) and (012) α-Ti. (purple).

**Figure 7:**
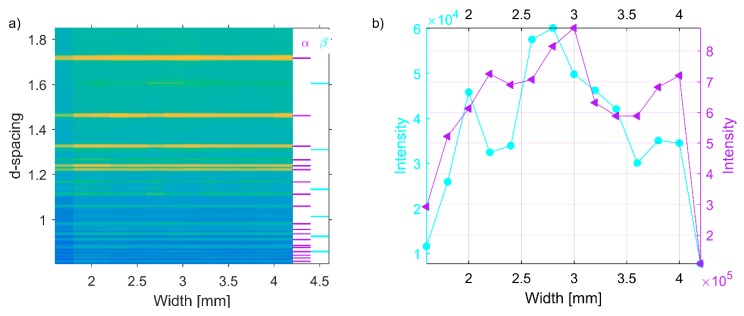
(**a**) Accumulated diffraction patterns of horizontal line scan along the build plane of specimen S8, and (**b**) corresponding peak intensity of (002) β-Ti (magenta) and (012) α-Ti (purple).

Similarly, diffraction patterns from one of the EBM Ti-6Al-4V specimens collected along the build direction and in the build plane are shown in Figure 6a and Figure 7a as a two-dimensional plot, viewed down the intensity axis, with the diffraction pattern number along the ordinate, and peak positions along the abscissa. For clarity and emphasizing the existence of β-Ti in the system, the peak intensity of well separated (002) β-Ti peak from the measurement presented in Figure 6a and Figure 7a is plotted in Figure 6b and Figure 7b, respectively. Peak intensity is colour coded to maintain consistency with previous figures. Note that the first and last data acquisition location (gauge volume) in Figure 7 does not exactly correspond to the edges of the specimen. There is about 300-μm clearance from each side. The peak intensity drop is shown in Figure 7b might be caused by the scan strategies used in this work. The outermost boundaries between the build and powder bed were melted first to form a contour. Thereafter powders inside the contour were melted in the manner of serpents’ path (from one side to another, then one step downwards followed by return, then one step downwards etc.), which is also known as hatching. As shown in Figure 5b and Figure 6, none of the diffraction patterns showed any sign of α’ martensite in the top layer of the build material unlike that reported in [15]. According to the Galarraga et al. [13], the observation of a martensitic structure is indicative of a high cooling rate imposed during solidification and subsequent cooling in the solid state of the last layer, and a cooling rate of >410 K/s may result in such a structure in last layer. Al-Bermani’s [15] and Galarraga et al. [13] have also reported that faster cooling rates after solution heat treatment produce a greater amount of α’ martensitic phase, with water-cooling at a rate of 650 K/s resulting in a fully α’ martensitic microstructure. However, apart from the last layer, the first layer can also vary in microstructure compared to the bulk, due to the faster cooling rate close to the thermal conductive substrate. And these pronounced effects of cooling rates on microstructure also render microstructural differences. Nonetheless, the volume of these microstructural extremes is relatively small. Interestingly, Galarraga et al. [13] did not observe α’ martensite in their unprocessed EBM built Ti-6Al-4V ELI (Extra Low Interstitial) specimens. However, based on the weakening of the (200) β phase diffraction peak at d-spacing = 1.60 Å and change in microstructure, tensile test results and microhardness values after post build heat treatment, they concluded that even air-cooled specimens may contain α’ martensite. The difference between the observation reported by Al-Bermani’s [15] and this study might be because of differences in specimen build geometry, process parameters and post-build heat treatments used in the two different studies.

During EBSD and SEM studies of EBM built Ti-6Al-4V, Al-Bermani et al. [15] observed 100% β-phase within the build height 0–300 μm and mostly α-phase at other parts. According to Al-Bermani et al. [15], the β phase occurs due to the co-melting and diffusion of the alloying elements in the austenitic stainless-steel base plate and the initial Ti-6Al-4V layers. The β phase stabilizing elements, such as Cr, Fe, and Ni were provided by melting of stainless-steel plate. Since all measurements presented in this work have been performed from 13 mm upwards, we are unable to confirm that observation. Nonetheless, based on observations presented in Figure 4, Figure 5, Figure 6 and Figure 7 it is safe to conclude that globally there is no significant phase difference in different parts of the EBM built Ti-6Al-4V parts at some distance away from the base plate despite the varied microstructure. This conclusion can be further supported by neutron diffraction studies. The deep penetration power into Ti and the larger sampling volume of neutrons provide the possibility to evaluate the bulk average phase compositions and residual stress/strain of all present phases in the system.

**Figure 8:**
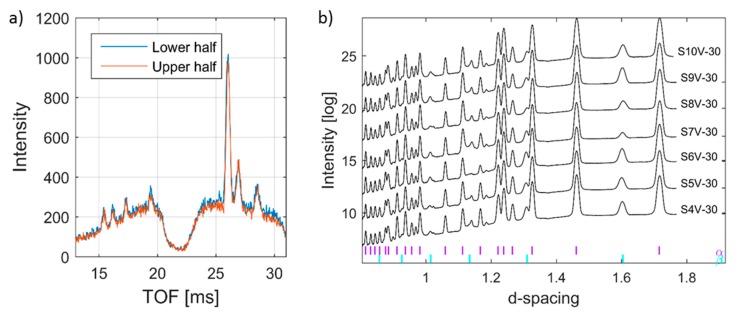
Time-of-flight neutron data for specimen S8 (**a**). Raw diffraction patterns depicting a relative comparison between the upper and lower half of specimen S7, supporting the observations in Figure 6. (**b**) Collection of diffraction patterns for seven different EBM Ti-6Al-4V specimens.

To cross-check for potential variations among larger sampling volumes, data sets collected at the ESS test beamline at HZB on the same specimen (S8) are presented in Figure 8a. The beamline operates in time of flight mode, where neutrons start to travel from a source at time t = 0 and travel tens of meters to the specimen at which point they have separated by their different velocities and hence wavelengths. In this measurement, an incident beam size of 10 mm × 10 mm was used, with the detector positioned at a scattering angle of 2θ = 90°. The results in Figure 8a present a relative direct comparison (the setup remained unchanged besides translating the specimen) between the upper half (red) and lower half (blue) of the specimen. Both diffraction patterns are almost identical to each other with the indication that there is no significant phase variation between the upper and lower part of the specimen. These results agree well with those in Figure 5b and supports the conclusion that phase distributions in the material are uniform.

**Figure 9:**
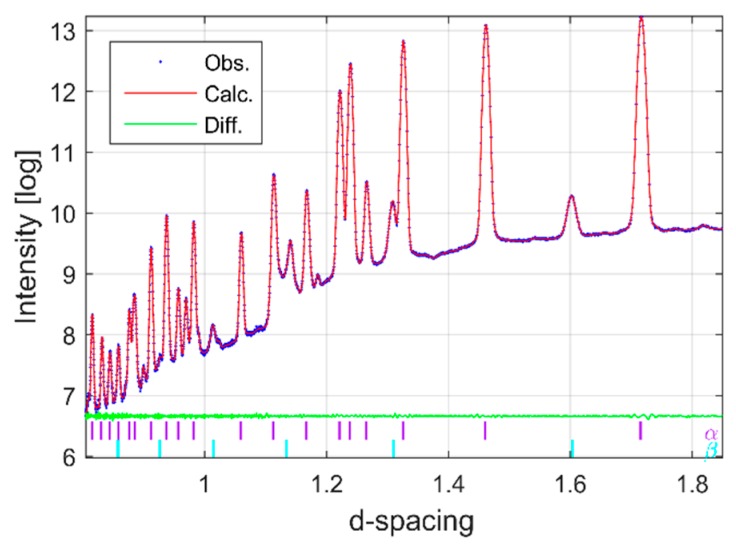
One typical Pawley refinement result: R_wp_ 1.73, Goodness of fit (GOF) 1.52.

To investigate whether different EBM process parameter combinations may lead to phase differences, an EDXRD pattern from the middle of different specimens is presented in Figure 8b. As shown in the figure, there is no phase difference between specimens other than intensity. As expected all specimens contain both α- and β-phases. Figure 9 represents a typical Pawley refinement result of data presented in Figure 6 and Figure 7. In that figure, the blue dots represent observed intensities, the red line represents the calculated, and the green line is the difference curve on the same scale. The color-coded tick marks indicate the calculated positions of Bragg peaks. As seen from the difference curve, the fit is highly satisfactory. All diffraction patterns contained some of the major α-Ti and β-Ti peaks, which are commonly reported in the literature [57] together with many other minor and high angle peaks which have not been included in any other reported studies. The matches of the interplanar spacings (d-spacings) obtained from these peaks and unit cell parameters of α-Ti and β-Ti were not in perfect agreement with the data of the ICSD (see, Table 2). This difference might be caused by that different EBM process parameters were used to manufacture the materials investigated in these studies, which may influence the lattice strains and hence the overall lattice parameter.

### 3.3. Residual Strain

**Figure 10:**
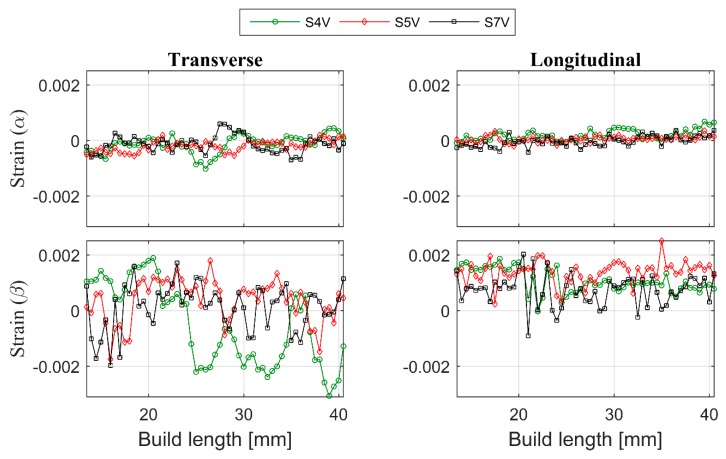
The lattice strain from the central vertical line of specimens perpendicular to the EBM build plane. Transverse direction at left and longitudinal direction at right. Color code of each specimen can be seen from the legend at top of the figure.

**Figure 11:**
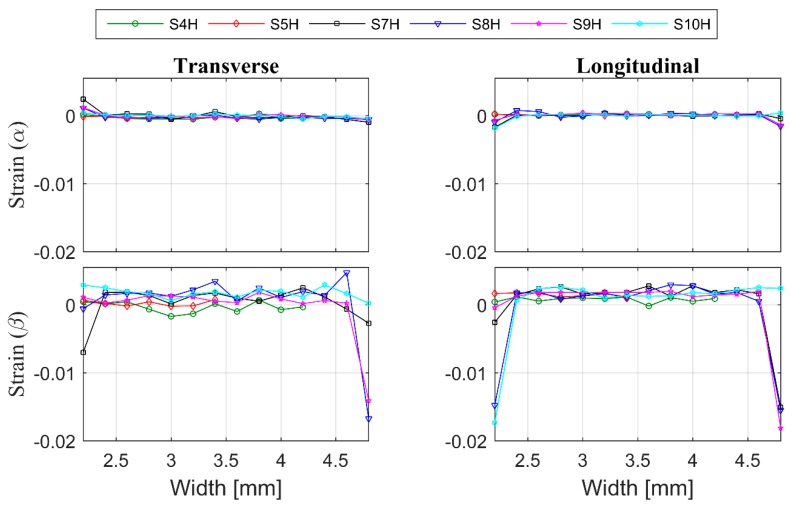
The lattice strain from the central horizontal line of a specimen that is parallel to the EBM build plane. Transverse direction at left, and longitudinal direction at right.

One of the biggest challenges in diffraction-based residual strain measurements of additively manufactured material is obtaining the strain-free reference lattice parameters. Commonly, the reference can be determined from a stress-free specimen of identical material. Because of the localized heating effect, microstructure variation and possible texture, specimens built by AM are expected to show some differences and even at different locations in the same specimen. Considering the microstructure difference between the ingredient powder, the corner of the build material and the center of the build material, only mean value-based strain calculations have been selected as the suitable strain reference extraction method. The lattice strain of α- and β-Ti calculated by using unit cell parameters (a and c) obtained from Pawley fitting of diffraction data from vertical and horizontal scans are shown in Figure 10 and Figure 11, respectively. Each coloured line in these figures corresponds to one specific scan belonging to a different specimen. The colour code is the same for both figures. The error bars are equal to or smaller than the marker size.

Recently, Tiferet et al. [27] have reported that none of SLM or EBM specimens contain residual macro- nor micro-strains, instead the ingredient powder contains residual strain. Interestingly, lattice parameters of α-Ti obtained from ingredient powder from our studies show almost identical result as their bulk specimen. In addition, in contrast to Tiferet et al. [27], lattice parameters obtained from specimen corners, ingredient powder and bulk specimens from our measurements are similar. Such small lattice parameter differences indicate that the residual strain in EBM built material is low. The difference between our results and Tiferet et al. [27] may be related to the different processing approaches that were used.

In addition to lattice strains calculated from unit cell parameters of α- and β-phases obtained from Pawley pattern fitting, a lattice specific strain was also calculated for selected α- and β-phase hkl reflections after single peak fitting. Despite the availability of other hkl reflections, only selected α-phase ((010), (012), (110), (004)), and β-phase ((011), (002), (211)) hkl reflections, which do not overlap or are well-separated from other phases, were used for calculation. The general trends of the strains from single peak-based calculations are similar to whole pattern fittings shown in Figure 10 and Figure 11. However, each lattice plane families yielded some disparity from one another. For simplicity and considering the superiority of strain calculation by using lattice parameters obtained by whole pattern fitting over single peak fitting, the results of single peak fitting are not shown.

From Figure 10, it is shown that the measured strain in both longitudinal and transverse directions in α- and β-Ti are fluctuating and they do not show any obvious trend with respect to deposition layers. However, closer inspection of the strain result of the α-Ti phase shows a slight compressive to tensile transition trend in both strain measurement directions (Figure 10) along the build. Even though the α-Ti strain result fluctuation does not match with the deposition layer, the frequency of the fluctuation appears to correlate with the length of the columnar grains. Therefore, to obtain reliable RS results from the experiment and modelling, we recommend considering the size of the primary β-grains in the measurement strategy, data interpretation and in theoretical simulations. Unlike α-Ti, the β-Ti did not show any observable transition and the strain appears to fluctuate more than the α-Ti (Figure 11). The reason for such a strong fluctuation might be that the β-Ti phase is the minority phase with high crystallographic symmetry and strong texture. Hence, the β-Ti phase in the Ti-6Al-4V system could be working as buffer phase, which causes local equilibrium of the stress/strain distribution in the system.

The material is built layer by layer via selectively melting the raw material. Therefore, each deposited layer in an additive manufactured structure undergoes multiple heating and cooling cycles with repetitive stress relaxation and accumulation processes. Hence, large thermal RS differences are to be expected at different heights in the build. However, at same build height or in same deposition layer, the heat treatment is expected to be the same. For that reason, the RS in the same layer should be the same. As predicted, the RS in the centre of the specimen measured along the scan direction showed linearity in both longitudinal and transverse directions (Figure 11). The magnitude and nature of the strain measured near the edge of most of the measurements appear similar to the result obtained from the centre or has a compressive nature.

**Figure 12:**
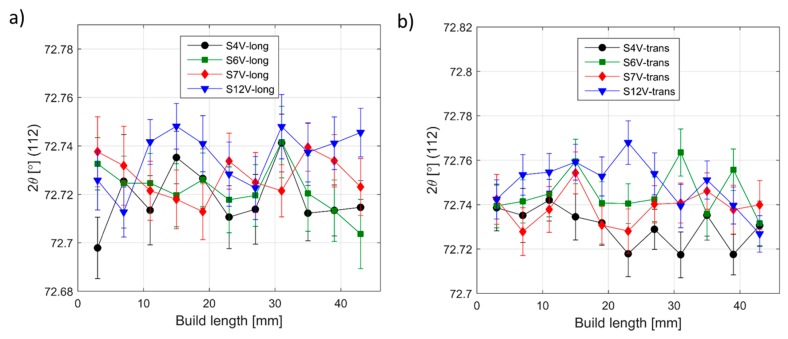
Variation of Ti(112) peak position with respect to build height by neutron diffraction. (**a**) Longitudinal direction, (**b**) transverse direction. It can be seen that variations are within the error bars.

The transverse strain in α-Ti, however, appears compressive on one side and tensile on the other. The EBM scan strategy used for building is expected to induce a sharp strain gradient from the surface/edge to the centre/inside of the build. During the EBM process, each layer of the target object is built in two steps. Firstly, the outer boundary is melted (contour). Then, in the second step, the actual part is built within the contours. The contour provides an interface between the actual build and the surrounding powder. Since molten metal is deposited on a colder contour wall, thermal contraction of the solidified material occurring during solidification creates a tensile stress in the deposit and compressive stress in the contour wall. The origin of this is not currently known. Whether it is build thickness or beam power requires further investigation. These observed differences within and between specimens may be attributed to the differences in their structure at the temperatures experienced during EBM processes. As shown in Figure 2a, the material microstructure consists of large prior β-grains with α-lamellas. Therefore, the scatter in the strain results presented could be understood to be a result of the grain statistics as the synchrotron X-ray experiment was conducted with a relatively small gauge volume. In order to justify whether this is the case, more measurements have been carried out on selected specimens with the E3 instrument at HZB. Similar to the synchrotron result, neutron diffraction measurements also did not show any significant difference from one another at different locations within the specimens (Figure 12). From Figure 12, one can see that there is neither any directional peak shift of the (112) peak of the α-Ti phase along the build direction nor peak position differences between specimens. This observation agrees with the results presented in Figure 10 and Figure 11. The main difference between the neutron diffraction results in Figure 12 and the X-ray diffraction results in Figure 10 and Figure 11 is that the neutron diffraction results seemed more linear than the X-ray diffraction results. This could indicate that there is indeed some grain size effect on the X-ray results. Therefore, to overcome similar problems, a larger beam size should be used in X-ray based measurements.

Residual stresses are important to control with regard to quality and integrity of parts built by AM. Therefore, it is crucial to know the status and magnitude of any residual stresses to be able to reduce any detrimental effects. Since residual stresses in additive manufactured materials arise mainly from temperature gradients, the stress levels formed in the parts could possibly be modified by changing the heat gradients during manufacturing. The scan speed, beam current and offset focus of the EBM process influences the heat gradients and are likely to cause different states of residual stresses. However, according to the present observations, the influence of these process parameters is not that obvious (at least not for the parameters selected in this work). The reason for not observing any strain/stress trends or the effects of different process parameters is most likely because of the high preheating temperature with slow cooling used in the EBM process. In SLM it was reported that heating the build platform ≥ 200 °C can reduce the RS levels [58]. Therefore, in the future RS studies, the role of the preheating temperature should be considered.

There are some limitations in the current work that could be addressed by future work, concerning process parameter diversity. In AM there are many processes parameters that affect the cooling rate of the build and consequently the microstructure and RS. Therefore, the authors propose that further investigations be performed with other processes parameters in mind. In Table 1 the process parameters that have been used in this work are shown. An example is that in this work, only a current of 8 mA has been used, this process parameter greatly affects the cooling rate and would undoubtedly render different RS results.

## 4. Conclusions

Residual strains in Ti-6Al-4V specimens built by AM from nominal 45–100 μm diameter gas atomised powder using an electron beam melting technique have been evaluated by using state-of-the-art neutron and synchrotron X-ray diffraction techniques. Based on the present studies of the EBM built Ti-6Al-4V materials, the following conclusions can be made:

1. Despite strong texture and a columnar microstructure in the build direction, a fairly uniform phase distribution has been observed. The microstructure is mostly Widmanstätten type with majority are α-Ti phase with some amount of β phase (1.5–10 wt %).
2. Based on synchrotron X-ray studies, longitudinal strain in α-Ti changes slightly from compression to tensile along the build direction, but no such trend is observed in β-Ti. Neutron studies showed no clear trend of RS in neither α- nor β-Ti.
3. Neither transverse nor longitudinal strains in α- and β-Ti in the build plane change significantly. The longitudinal strains in both α- and β-Ti, however, showed a compressive nature close to the edges of the built materials.
4. No clear residual strain differences between deposition layers was found.
5. The strain variation along the build direction may be related to the elongated prior β-grains and should be considered in future experimentation and analysis.

## Figures and Tables

**Table 1:** Specimen process parameters.

Specimen ID	Contour Scan Speed (mm/s)	Scan Speed (mm/s)	Current (mA)	Contour Current (mA)	Offset Focus (mA)	Specimen Thickness (mm)
S4	180	575	8	9	15	2.47/5 *
S5	180	650	8	9	15	2.68
S6	180	651	8	9	15	2.70/5 *
S7	180	652	8	9	15	1.99/5 *
S8	180	575	8	9	30	3.02
S9	180	652	8	9	30	2.93
S10	180	653	8	9	30	2.77
S11	180	650	8	9	30	2.97
S12	180	700	8	9	12	2.50/5 *

* is the as-built specimen thickness that was used in the neutron diffraction studies.

**Table 2:** Some known and calculated crystallographic information of different Ti phases.

Phase	Structure	Space Group	*a*(Å)	*c*(Å)	(°C)	Atomic Site	Reference (ICSD)
α(Ti)	HCP	P63/mmc	2.9511	4.6843	25	0.3333 0.6667 0.25	43416
			2.9508	4.6855	-		52522
			2.9064	4.6667	20		99778
			2.951	4.682	-		43614
			2.916	4.631	-		168830
			2.9232(7)	4.6700(4)	25		Polycrystal
			2.9230(6)	4.6697(6)	25		Polycrystal corner
			2.9210(6)	4.6644(8)	25		Powder
β(Ti)	BCC	$Im\bar{3}m$	3.2765	3.2765	-	000	653278
			3.2068(7)	3.2068(7)	25		Polycrystal
			3.2082(7)	3.2082(7)	25		Polycrystal corner
			3.2023(8)	3.2023(8)	25		Powder
Omega Ti	Loose HCP	P6/mmm	4.6	2.82	High pressure	Ti1 000; Ti2 0.3333 0.6667 0.5	52521

## References

[1] Gerd L., James C.W. (2007). Titanium.

[2] Safdar A., He H.Z., Wei L., Snis A., Chavez d.P. (2012). Effect of process parameters settings and thickness on surface roughness of EBM produced Ti-6Al-4V. Rapid Prototyp. J..

[3] Murr L.E., Esquivel E.V., Quinones S.A., Gaytan S.M., Lopez M.I., Martinez E.Y., Medina F., Hernandez D.H., Martinez E., Martinez J.L. (2009). Microstructures and mechanical properties of electron beam-rapid manufactured Ti-6Al-4V biomedical prototypes compared to wrought Ti-6Al-4V. Mater. Charact..

[4] Murr L.E., Quinones S.A., Gaytan S.M., Lopez M.I., Rodela A., Martinez E.Y., Hernandez D.H., Martinez E., Medina F., Wicker R.B. (2009). Microstructure and mechanical behavior of Ti-6Al-4V produced by rapid-layer manufacturing, for biomedical applications. J. Mech. Behav. Biomed. Mater..

[5] Tan X., Kok Y., Tan Y.J., Descoins M., Mangelinck D., Tor S.B., Leong K.F., Chua C.K. (2015). Graded microstructure and mechanical properties of additive manufactured Ti-6Al-4V via electron beam melting. Acta Mater..

[6] Neikter M., Åkerfeldt P., Pederson R., Antti M.-L., Sandell V. (2018). Microstructural characterization and comparison of Ti-6Al-4V manufactured with different additive manufacturing processes. Mater. Charact..

[7] Zhang L., Liu Y., Li S., Hao Y. (2018). Additive Manufacturing of Titanium Alloys by Electron Beam Melting: A Review. Adv. Eng. Mater..

[8] Liu Y.J., Wang H.L., Li S.J., Wang S.G., Wang W.J., Hou W.T., Hao Y.L., Yang R., Zhang L.C. (2017). Compressive and fatigue behavior of beta-type titanium porous structures fabricated by electron beam melting. Acta Mater..

[9] Liu Y.J., Li S.J., Wang H.L., Hou W.T., Hao Y.L., Yang R., Sercombe T.B., Zhang L.C. (2016). Microstructure, defects and mechanical behavior of beta-type titanium porous structures manufactured by electron beam melting and selective laser melting. Acta Mater..

[10] Zhao S., Li S.J., Wang S.G., Hou W.T., Li Y., Zhang L.C., Hao Y.L., Yang R., Misra R.D.K., Murr L.E. (2018). Compressive and fatigue behavior of functionally graded Ti-6Al-4V meshes fabricated by electron beam melting. Acta Mater..

[11] Safdar A., Wei L.-Y., Snis A., Lai Z. (2012). Evaluation of microstructural development in electron beam melted Ti-6Al-4V. Mater. Charact..

[12] Neikter M., Woracek R., Maimaitiyili T., Scheffzük C., Strobl M., Antti M.-L., Åkerfeldt P., Pederson R., Bjerkén C. (2018). Alpha texture variations in additive manufactured Ti-6Al-4V investigated with neutron diffraction. Addit. Manuf..

[13] Galarraga H., Warren R.J., Lados D.A., Dehoff R.R., Kirka M.M., Nandwana P. (2017). Effects of heat treatments on microstructure and properties of Ti-6Al-4V ELI alloy fabricated by electron beam melting (EBM). Mater. Sci. Eng. A.

[14] de Formanoir C., Michotte S., Rigo O., Germain L., Godet S. (2016). Electron beam melted Ti-6Al-4V: Microstructure, texture and mechanical behavior of the as-built and heat-treated material. Mater. Sci. Eng. A.

[15] Al-Bermani S.S., Blackmore M.L., Zhang W., Todd I. (2010). The Origin of Microstructural Diversity, Texture, and Mechanical Properties in Electron Beam Melted Ti-6Al-4V. Metall. Mater. Trans. A.

[16] Fitzpatrick M.E., Lodini A. (2003). Analysis of Residual Stress by Diffraction using Neutron and Synchrotron Radiation.

[17] Yang X., Richard Liu C. (1999). Machining titanium and its alloys. Mach. Sci. Technol..

[18] Hauk V., Hauk V. (1997). Structural and Residual Stress Analysis by Nondestructive Methods. Structural and Residual Stress Analysis by Nondestructive Methods.

[19] Withers P.J., Bhadeshia H.K.D.H. (2001). Residual stress. Part 2—Nature and origins. Mater. Sci. Technol..

[20] Szost B.A., Terzi S., Martina F., Boisselier D., Prytuliak A., Pirling T., Hofmann M., Jarvis D.J. (2016). A comparative study of additive manufacturing techniques: Residual stress and microstructural analysis of CLAD and WAAM printed Ti–6Al–4V components. Mater. Des..

[21] Shi X., Ma S., Liu C., Wu Q., Lu J., Liu Y., Shi W. (2017). Selective laser melting-wire arc additive manufacturing hybrid fabrication of Ti-6Al-4V alloy: Microstructure and mechanical properties. Mater. Sci. Eng. A.

[22] Ali H., Ma L., Ghadbeigi H., Mumtaz K. (2017). In-situ residual stress reduction, martensitic decomposition and mechanical properties enhancement through high temperature powder bed pre-heating of Selective Laser Melted Ti6Al4V. Mater. Sci. Eng. A.

[23] Mishurova T., Cabeza S., Artzt K., Haubrich J., Klaus M., Genzel C., Requena G., Bruno G. (2017). An Assessment of Subsurface Residual Stress Analysis in SLM Ti-6Al-4V. Materials.

[24] Yadroitsev I., Yadroitsava I. (2015). Evaluation of residual stress in stainless steel 316L and Ti6Al4V samples produced by selective laser melting. Virtual Phys. Prototyp..

[25] Hrabe N., Gnäupel-Herold T., Quinn T. (2017). Fatigue properties of a titanium alloy (Ti–6Al–4V) fabricated via electron beam melting (EBM): Effects of internal defects and residual stress. Inter. J. Fatigue.

[26] Vastola G., Zhang G., Pei Q.X., Zhang Y.-W. (2016). Controlling of residual stress in additive manufacturing of Ti6Al4V by finite element modeling. Addit. Manuf..

[27] Tiferet E., Rivin O., Ganor M., Ettedgui H., Ozeri O., Caspi E.N., Yeheskel O. (2016). Structural investigation of selective laser melting and electron beam melting of Ti-6Al-4V using neutron diffraction. Addit. Manuf..

[28] Withers P.J., Bhadeshia H.K.D.H. (2001). Residual stress. Part 1—Measurement techniques. Mater. Sci. Technol..

[29] Maimaitiyili T., Bjerken C., Steuwer A., Wang Z., Daniels J., Andrieux J., Blomqvist J., Zanellato O. (2017). In situ observation of γ-ZrH formation by X-ray diffraction. J. Alloy. Compd..

[30] Maimaitiyili T., Steuwer A., Blomqvist J., Bjerkén C., Blackmur M.S., Zanellato O., Andrieux J., Ribeiro F. (2016). Observation of the δ to ε Zr-hydride transition by in-situ synchrotron X-ray diffraction. Cryst. Res. Technol..

[31] Maimaitiyili T., Blomqvist J., Steuwer A., Bjerkén C., Zanellato O., Blackmur M.S., Andrieux J., Ribeiro F. (2015). In situ hydrogen loading on zirconium powder. J. Synchrotron Radiat..

[32] Withers P.J., Preuss M., Steuwer A., Pang J.W.L. (2007). Methods for obtaining the strain-free lattice parameter when using diffraction to determine residual stress. J. Appl. Crystallogr..

[33] Korsunsky A.M., Song X., Hofmann F., Abbey B., Xie M., Connolley T., Reinhard C., Atwood R.C., Connor L., Drakopoulos M. (2010). Polycrystal deformation analysis by high energy synchrotron X-ray diffraction on the I12 JEEP beamline at Diamond Light Source. Mater. Lett..

[34] Wimpory R.C., Mikula P., Šaroun J., Poeste T., Li J., Hofmann M., Schneider R. (2008). Efficiency Boost of the Materials Science Diffractometer E3 at BENSC: One Order of Magnitude Due to a Horizontally and Vertically Focusing Monochromator. Neutron News.

[35] Coelho A. TOPAS Academic: Technical Reference. TOPAS Academic: Technical Reference 2004, V4.1. http://www.TOPAS-academic.net/.

[36] Neikter M. (2018). Microstructure and Texture of Additive Manufactured Ti-6Al-4V. Licentiate Thesis.

[37] Cansizoglu O., Harrysson O.L.A., West H.A., Cormier D.R., Mahale T. (2008). Applications of structural optimization in direct metal fabrication. Rapid Prototyp. J..

[38] Algardh J.K., Horn T., West H., Aman R., Snis A., Engqvist H., Lausmaa J., Harrysson O. (2016). Thickness dependency of mechanical properties for thin-walled titanium parts manufactured by Electron Beam Melting (EBM)^®^. Addit. Manuf..

[39] Karlsson J., Snis A., Engqvist H., Lausmaa J. (2013). Characterization and comparison of materials produced by Electron Beam Melting (EBM) of two different Ti–6Al–4V powder fractions. J. Mater. Process. Technol..

[40] Electron Beam Melting. http://www.arcam.com/technology/electron-beam-melting/2016.

[41] Wang X., Gong X., Chou K. (2015). Scanning Speed Effect on Mechanical Properties of Ti-6Al-4V Alloy Processed by Electron Beam Additive Manufacturing. Procedia Manuf..

[42] Qian L., Mei J., Liang J., Wu X. (2005). Influence of position and laser power on thermal history and microstructure of direct laser fabricated Ti–6Al–4V samples. Mater. Sci. Technol..

[43] Klingbeil N.W., Beuth J.L., Chin R.K., Amon C.H. (2002). Residual stress-induced warping in direct metal solid freeform fabrication. Inter. J. Mech. Sci..

[44] Cullity B.D., Stock S.R. (2013). Elements of X-ray Diffraction.

[45] Strobl M., Bulat M., Habicht K. (2013). The wavelength frame multiplication chopper system for the ESS test beamline at the BER II reactor—A concept study of a fundamental ESS instrument principle. Nucl. Instrum. Methods Phys. Res. A.

[46] Woracek R., Hofmann T., Bulat M., Sales M., Habicht K., Andersen K., Strobl M. (2016). The test beamline of the European Spallation Source—Instrumentation development and wavelength frame multiplication. Nucl. Instrum. Methods Phys. Res. A.

[47] Belsky A., Hellenbrandt M., Karen V.L., Luksch P. (2002). New developments in the Inorganic Crystal Structure Database (ICSD): Accessibility in support of materials research and design. Acta Crystallogr. B Struct. Sci..

[48] Daymond M.R., Bourke M.A.M., Dreele R.B.V. (1999). Use of Rietveld refinement to fit a hexagonal crystal structure in the presence of elastic and plastic anisotropy. J. Appl. Phys..

[49] Lundbäck A., Pederson R., Colliander M.H., Brice C., Steuwer A., Heralic A., Buslaps T., Lindgren L., Venkatesh V., Pilchak A.L., Allison J.E., Ankem S., Boyer R., Christodoulou J., Fraser H.L., Imam M.A., Kosaka Y., Rack H.J. (2016). Modeling And Experimental Measurement with Synchrotron Radiation of Residual Stresses in Laser Metal Deposited Ti-6Al-4V. Proceedings of the 13th World Conference on Titanium.

[50] Liu S., Shin Y.C. (2019). Additive manufacturing of Ti6Al4V alloy: A review. Mater. Des..

[51] Kelly S.M., Kampe S.L. (2004). Microstructural evolution in laser-deposited multilayer Ti-6Al-4V builds: Part I. Microstructural characterization. Metall. Mater. Trans. A.

[52] Zhao X., Li S., Zhang M., Liu Y., Sercombe T.B., Wang S., Hao Y., Yang R., Murr L.E. (2016). Comparison of the microstructures and mechanical properties of Ti–6Al–4V fabricated by selective laser melting and electron beam melting. Mater. Des..

[53] Da Silva S.L.R., Kerber L.O., Amaral L., dos Santos C.A. (1999). X-ray diffraction measurements of plasma-nitrided Ti–6Al–4V. Surf. Coat. Technol..

[54] Xu W., Lui E.W., Pateras A., Qian M., Brandt M. (2017). In situ tailoring microstructure in additively manufactured Ti-6Al-4V for superior mechanical performance. Acta Mater..

[55] Murty S.V.S.N., Nayan N., Kumar P., Narayanan P.R., Sharma S.C., George K.M. (2014). Microstructure–texture–mechanical properties relationship in multi-pass warm rolled Ti–6Al–4V Alloy. Mater. Sci. Eng. A.

[56] Chen L.Y., Huang J.C., Lin C.H., Pan C.T., Chen S.Y., Yang T.L., Lin D.Y., Lin H.K., Jang J.S.C. (2017). Anisotropic response of Ti-6Al-4V alloy fabricated by 3D printing selective laser melting. Mater. Sci. Eng. A.

[57] Muguruma T., Iijima M., Brantley W.A., Yuasa T., Ohno H., Mizoguchi I. (2011). Relationship between the metallurgical structure of experimental titanium miniscrew implants and their torsional properties. Eur. J. Orthod..

[58] Mercelis P., Kruth J.-P. (2006). Residual stresses in selective laser sintering and selective laser melting. Rapid Prototyp. J..

